# On Consecutive Syphilis

**Published:** 1843-12-30

**Authors:** Meredith Clymer


### THE MEDICAL EXAMINER,

### And Retrospect of the Medical Sciences.

Vol. VI.] PHILADELPHIA, SATURDAY, DECEMBER 30, 1843. [No. 26.

### CLINICAL LECTURES AND REPORTS.

### CLINIC OF THE UNIVERSITY OF PENNSYLVANIA AT THE PHILADELPHIA HOSPITAL.

BY MEREDITH CLYMER, M. D.

(Reported for the Medical Examiner.)

LECTURE II.

### ON CONSECUTIVE SYPHILIS.

*Wednesday, Dec. 6th.* GENTLEMEN:—In my last lecture I spoke of the local treatment of chancre. I wish to-day to make some remarks on the general or specific treatment of venereal sores. A great diversity of opinion has prevailed of late years in the profession, in regard to the administration of mercury in primary venereal affections. Its utility has been questioned or denied by one class of practitioners, and positive harm is said by others to follow its use. Some enthusiasts have gone so far as to assert that all the morbid phenomena known as consecutive or secondary syphilis were due to mercury, and did not result from the disease. Some practitioners, on the other hand, rely exclusively on mercury for the treatment of every sore on the genitals. Now, I will endeavour to show you under what circumstances mercury should be employed in primary syphilis, and with what restrictions.

You have seen that at first syphilis is a purely local affection, that the action of the virus is limited to the infected spot, and that the system is uncontaminated. The chances of constitutional contamination are in direct proportion to the length of time the first stage of chancre, or that of ulceration, exists. Dr. Ricord has never seen secondary syphilis where the primary sore was radically healed within five days. I say *radically healed*, because the cicatrisation of a chancre is often illusory; a chancre may apparently be healed perfectly, yet, on examination, you will find the cicatrix indurated; whenever this state of things exist, and it is very common after the simple treatment of chancre, you may be certain that the disease is there, and that, moreover, the constitution is implicated. In such cases the cicatrix is very obnoxious to ulceration, and a genuine chancre is the consequence. Induration may be considered, as I told you, as the index of the absorption of the venereal virus, for the indurated chancre is always followed by constitutional symptoms. Indurated chancres are, as we have seen, indolent—show no disposition to spread, or to heal; they may remain for months perfectly stationary. We have, then, in the induration, a guide for the administration of mercury—a hydrargyrometer. Whenever there is a chancre with hard callous edges and base, you should give mercury; whenever an indurated cicatrix exists after the healing of a chancre, a mercurial treatment is required; and finally, where no induration exists, but where, after a fair trial of the simple treatment, the sore will not heal, you had better employ mercury. Does mercury diminish the risk of consecutive syphilis? There is a vast mass of evidence, I think, to prove conclusively that it does. From numerous statistical inquiries it would seem that secondary symptoms occur about 1 in 10 times where no mercury has been administered, and only 1 in 75 where this remedy has been resorted to. Whilst, therefore, most intelligent surgeons of the present day condemn an indiscriminate mercurial treatment, they still regard mercury as a certain and powerful remedial agent that can be employed in the majority of cases, both of primary and consecutive syphilis.

I have pointed out to you in what instances mercury is to be employed in the treatment of primary syphilis, I will now endeavour to show you where it is inadmissible. Mercury should never be given where a phagadenic sore exists; it will almost invariably increase the danger and sufferings of the patient. It was the administration of the drug in this form of the disease that brought so much discredit upon it amongst the army surgeons and others, and produced so many partisans of the simple treatment. This class of venereal sores I have described to you; their treatment, which is very perplexing, and but too often unsuccessful, I shall make the subject of a special lecture at a future period, when I can illustrate my remarks with specimens of the disease. Another contraindication to the employment of mercury in chancre is the existence of inflammation in the sore. If the sore be inflamed or very irritable, its condition will be aggravated by mercury, and the constitution of the patient will suffer too. Mercury should not, as a general rule, to which there are hardly any exceptions, be employed in the ulcerative stage of chancre; it will do harm. The introduction of the simple or non-mercurial treatment in syphilis, whatever may be its real therapeutic value, has contributed greatly to improve our practice, and render it more rational and safe; it has led to the study and investigation of the natural history of the disease, and it has assigned to mercury an absolute, positive value; it has shown us where it is admissible and where not; and in what conditions of the system its use should be abstained from. If there be derangement of the stomach and bowels, you should correct it before mercurialising. A suitable regimen will materially assist your specific treatment. Let your patient have a light, nourishing diet; let him abstain from rich or highly seasoned food, but, on the other hand, do not starve him. Milk, light meats and broths, may be allowed according to circumstances. Forbid the use of alcoholic and malt liquors. A small quantity of Madeira or Sherry wine in water, or very weak brandy and water, however, is sometimes beneficial. The mercurial employed may be either blue-pill, calomel, corrosive sublimate, or the iodide of mercury; they should all be combined with some preparation of opium. I usually employ the proto-iodide of mercury, combined with lactucarium and the extracts of opium and cicuta. I give from one to two grains of the iodide, in divided doses, in the course of the day. I rarely find it to disagree with the stomach or bowels. If you direct the Castillon powders at breakfast and tea, the patient will generally escape diarrhœa. If there be contraindications to the internal use of the medicine, you may use the mercurial ointment endermically on the insides of the thighs and arms; or have recourse to mercurial fumigations with cinabar, about two drachms of which may be employed each time. But where, under suitable precautions, you use the iodide, you will find it, I think, the best preparation.

You should never, if possible, induce salivation in treating chancre. It complicates the disease, retards the treatment, and injures the patient's constitution. On its subsidence you will find the disease pretty much where you commenced. A new affection has been set up, and on its disappearance the old one returns. Dr. Graves relates a case of syphilitic iritis which he salivated profusely at its commencement, but without success; but which subsequently recovered under a judicious mercurial course. The susceptibility of individuals to the effects of mercury varies greatly, so that no absolute rule as to quantity can be given; it must be relative. A small quantity affects some, whilst others, again, will bear large doses of the drug without any apparent result. Begin with small doses, and gradually increase them until some positive result occurs, unless mercurial accidents should supervene; and in that case stop immediately. If, however, you give small doses, and watch their effect, and as soon as the mouth gives evidence of the mercurial action, diminish the dose, you will be generally enabled to pursue your treatment without interruption until a cure is effected. How long are we to continue the exhibition of mercury? Much difference of opinion prevails on this point. The late Professor Dubois used to say, that if a man had a chancre at eighteen and died at eighty, he should always, to the day of his death, make it a rule to take a mercurial pill daily before breakfast, as a *hors d'œuvre*. Dupuytren continued the mercurial treatment, after a complete cure had been effected, for the same length of time that it had taken to dissipate the original symptoms. Chomel continues the treatment for five or six months; a less time he does not consider safe. Ricord gives mercury from four to six weeks after the cure, gradually diminishing the quantity. Our guide should be its effects on the local phenomena; when these have disappeared, and no general symptoms manifest themselves, then the remedy is no longer called for.

When the system becomes infected a new train of phenomena occurs, which is called consecutive or constitutional syphilis, or secondary symptoms. We have numerous cutaneous eruptions; inflammation and ulceration of the palate and fauces; inflammation of the eye; induration and ulceration of the glands of the skin; inflammation of the osseous and fibrous tissues,—in fine, although the syphilitic virus when it has entered the system shows an elective affinity for some tissues, to the comparative neglect of others, still there are no parts of the frame but may feel its influence. Cases of syphilitic disease of the brain are recorded by Ricord and others; and many of the severest forms of neuralgia are doubtless due to this cause.

### SYPHILITIC CUTANEOUS AFFECTIONS.

Constitutional syphilis may assume the form of any of the eruptions of the skin, but some are more constantly met with than others. They are sometimes concurrent with primary symptoms, and then their diagnosis is easy; but they more frequently appear at a remote period, and then it is more difficult. Syphilitic eruptions have certain general characters, which are by many considered as pathognomonic, by which they may be distinguished from ordinary skin-diseases. The most frequent and positive of these is the peculiar dusky livid hue, or copper colour of the eruption. This sign varies very much. Although a practised eye will frequently recognise it, it is not always present, and if relied on solely, will often mislead. A circular form, or tendency to it, in the eruptive patches, has also been given as a character of the syphilides, but I do not know that it is more common in this variety of eruptions than in any other. The consecutive ulcerations are peculiar, and not to be mistaken—they are deeply excavated, round, with callous perpendicular edges; are often serpiginous, describing spirals, or the segments of circles; and are covered by thick greenish or black scabs. On the disappearance of the eruptions a dark stain commonly remains on the skin for some time; or indelible white lineal, or spiral cicatrices. Itching is a very rare accompaniment in syphilitic eruptions, being generally absent. A peculiar repulsive odour is frequently exhaled. The parts of the body most exposed are those most commonly affected—as the face, forehead, shoulders, &c.

[Various cases of syphilitic eruptions were shown to the class and described. A case of squamous eruption on the breast and shoulders, with tubercular pustules on the forehead. A case of rupia in a female. A case of tubercular eruption on the face, with iritis, &c. &c.]

Now, how should we treat syphilitic eruptions? Our chief reliance must be on mercury. I know of no other remedy which will effect a radical cure. You may get rid of the eruption by other means, local and general, but it will invariably return. In the case of the woman with the scabby eruption on the breast, and syphilitic tubercles on the forehead, no mercury was given either for the primary symptoms, or for the first attack of the syphilitic eruption, nine months since, and the consequence is a return of the disease. I treated a female last summer for syphilitic psoriasis, whom I cured of a primary affection six years since by the simple treatment. She was opposed violently to mercury, and I gave her the iodide of potassium, and the eruption disappeared in a short time under its use. It reappeared again in a month or two. I tried Donovan's Solution, and persevered in its use for some time, having just before treated a case of simple psoriasis successfully with it; but its influence was very slight; I then resorted to mercury, and speedily cured her. I give the iodide of mercury in the manner I have just indicated to you—or the bichloride in doses of the tenth of a grain. When there are ulcerations you must employ local measures, and your success will often depend on the variety of your resources. Sometimes the solid nitrate of silver, or the sulphate of copper will hasten cicatrisation; sometimes a weak solution of the chloride of zinc; the iodide of mercury in honey (ʒj. to ℥iss.) is an excellent local application, as is powdered calomel sprinkled on the ulcer; the aromatic wine alone, or combined with tannin and very dilute nitric acid lotion, are often highly successful.

### NODES AND SYPHILITIC RHEUMATISM.

Nodes are the result of superficial inflammation of the bones and their fibrous coverings, terminating in an effusion or deposit between the bones and the periosteum. Venereal disease of the bones and periosteum may sometimes be mistaken for simple rheumatic periostitis. The history of the case, and the aspect of the patient, will materially assist us in forming a differential diagnosis. In rheumatic periostitis, the seat of pain is more frequently the joints and fleshy parts of the limb. Whereas, in syphilitic periostitis, the shafts of the long bones and the bones of the head are most frequently affected. Nodes terminate either by resolution with absorption of the effused matter, or else by ulceration and caries of the bone. The rheumatic and neuralgic pains of constitutional syphilis, generally accompany the disease of the osseous system; but may exist independently of it. Collateral evidence must assist us in making out our diagnosis.

Before speaking of the general treatment of nodes, I will mention the local. If there be much inflammation, rest and antiphlogistic measures must be employed. Leeches may be applied around the tumour; and use local applications calculated to remove the pain and inflammation. Do not open a node, even when fluctuation is present. Exfoliation will follow, which will be very tedious, and often give rise to deformity. If the distension be very great, and there is no other means of relieving the suffering, draw off the fluid with a fine trochar. Under ordinary circumstances rely on the general treatment and blisters. In chronic nodes repeated blisters, the strong tincture of iodine, and mercurial ointment are the best local means we can employ.

Mercury was until lately used as the specific treatment in this form of syphilis. Within a few years, the iodide of potassium has been employed with great success, and has generally superseded mercury. In the syphilitic affections of the osseous fibrous tissues, and in deep seated cellular tubercles, I believe it to be better than mercury. I have seen it extensively used in this class of affections by Dr. Ricord and others, and have employed it myself within this house, and in private practice, and with invariable success. By some practitioners it is given in enormously large doses, but without any corresponding results. I usually give five grains of the salt, three times a day in hop tea, or sweetened water, sometimes increasing the dose. If cerebral or gastric uneasiness supervene, I intermit its use for a day or two. Under its use patients frequently grow fat, the appetite improves, and all their secretions become regular. It sometimes produces severe coryza, which disappears, however, in the course of a day. Salivation is said to follow its use; this accident I have never seen. A good nutritous diet, and regular exercise and fresh air, are very essential to patients, whose constitution is infected with syphilis. These you will find powerful adjuvants in the treatment.

I have spoken of Donovan's solution. This is the liquor hydriodatis hydrargyri et arsenici, a new remedy which is occupying the attention of physicians a good deal. It has been highly recommended in the treatment of constitutional syphilis. I introduced it into this house last summer, and it has since been very extensively used. I have also used it in private practice. In syphilitic cutaneous affections, and in nodes, I have derived no benefit whatever from it. In cases of chronic rheumatism, which might have been syphilitic, indeed where in the majority the presumption was very strong, amounting almost to certainty, it was generally successful; so rapidly often as to leave much doubt in my mind, whether the cure was due to the remedy, or to the spontaneous subsidence of the disease, the result of improved hygienic relations. One fatal case occurred under its use. This was an old woman, suffering under chronic rheumatism, particularly of the wrist joints; the medicine was given in five drop doses three times a day in water, and the affected joints were painted twice daily with a strong tincture of iodine. The disease rapidly subsided, but gastro-enteric symptoms occurred; the medicine was immediately discontinued, but the patient died; the intestines were found to be ulcerated. I do not mean to attribute the death in the present instance to the remedy, but I merely mention the fact to you. I have seen gastric symptoms frequently occur during its use, but they have in all other instances subsided on the intermission of the remedy and the administration of diluents. Remember then, that you have to deal with a very powerful remedy, and be cautious in its exhibition.

### SYPHILITIC IRITIS.

I shall next exhibit to you a case of syphilitic iritis. The iris of all the tissues of the eye, is thought to be most liable to the effects of constitutional syphilis. But in the cases of inflammation of the iris in syphilitic individuals which I have seen, the other tissues of the eye were compromised along with the iris. Dr. Graves thinks that the disease always commences in the mucous tissue, and subsequently extends to the others, and that it should be called syphilitic ophthalmia.

Now what are the symptoms by which we recognize iritis in this man? If you will look at his eye, you will find the pupil very much contracted, and the aperture somewhat angular; on letting a strong light shine on it, no mobility is observable. Its edges are thickened, with a tendency to inversion, and are irregular. The colour of the iris is changed; it is of a much deeper hue than natural, and evidently thickened. On looking attentively through the pupil you will perceive a white flaky substance; this is coagulable lymph, on the anterior capsule of the crystalline lens, the result of the extension of the inflammation to that tissue. The vision of this man is seriously impaired, and he has a good deal of pain in the region of the orbit, especially at night. There is blepharitis, and ocular conjunctivitis, together with slight keratitis, the cornea being somewhat cloudy. Probably the whole organ is more or less affected. There is great intolerance of light, and there is some difficulty in exposing the eye.

The syphilitic nature of the disease we infer from the history of the case; the man having had primary sores about three years ago, and frequent gonorrhœas since, which may or may not have been accompanied with chancres, and by the presence of a well marked tubercular syphilitic eruption, which covers his face, and neck, and back.

What is the treatment of syphilitic iritis? Mercury is our sheet anchor. We must induce its special effects on the system as speedily as possible, but we must avoid profuse salivation, for, as in the case related by Dr. Graves, in his Clinical Lectures, the disease may progress in spite of it. If the symptoms are urgent give five grains of calomel combined with an opiate two or three times a day, or a larger quantity of blue mass; and if the individual is not readily susceptible to mercury, rub mercurial ointment into the thighs or arms, or under the jaw. An active cathartic should ordinarily commence the treatment, acting as a revulsive and evacuant, and at the same time increasing the activity of the absorbents, and hastening the mecurial action. If the patient is stout, and the inflammation is high employ venesection, and repeat it, if circumstances will permit. Too frequently, however, the syphilitic cachexia is so strongly marked as to forbid general depletion. You must then resort to cupping on the back of the neck, or to leeching at the temples, and beneath the infra-orbitar ridge. A blister applied to the back of the neck on the decline of the disease will often facilitate its resolution. A most important part of the treatment is to smear over the eye-brow and temple of the affected side with extract of belladonna and mild mecurial ointment, equal parts of each. This should be applied twice a day; it serves to prevent adhesion of the iris, and assuages the orbitar pain, which is often so intolerant at night. Sichel is of opinion that the action of the belladonna, is also directly antiphlogistic. I can only conceive it to be so on the grounds of its controlling the circulation by its action on the branches of the sympathetic distributed to the eye, and not from the mechanical explanation he offers. Although local applications are of no avail in simple iritis, yet in compound affections much assistance will be afforded by their use. I shall therefore in addition to the specific treatment directed to the iritis, order the usual local treatment for the conjunctivitis.

